# The impact of body mass index (BMI) on MRI diagnostic performance and surgical management for axillary lymph node in breast cancer

**DOI:** 10.1186/s12957-022-02520-6

**Published:** 2022-02-23

**Authors:** Shu-Tian Chen, Hung-Wen Lai, Wen-Pei Wu, Shou-Tung Chen, Chiung-Ying Liao, Hwa-Koon Wu, Dar-Ren Chen, Chi Wei Mok

**Affiliations:** 1 Department of Diagnostic Radiology, Chang Gung Memorial Hospital-Chiayi Branch, Chiayi, Taiwan; 2 Chang Gung University College of Medicine, Taoyuan City, Taiwan; 3 Department of Biomedical Imaging and Radiological Sciences, National Yang Ming Chiao Tung University, Taipei, Taiwan; 4 Endoscopy & Oncoplastic Breast Surgery Center, Changhua Christian Hospital, Changhua, Taiwan; 5 Division of General Surgery, Changhua Christian Hospital, Changhua, Taiwan; 6 Comprehensive Breast Cancer Center, Changhua Christian Hospital, Changhua, Taiwan; 7 Minimal Invasive Surgery Research Center, Changhua Christian Hospital, Changhua, Taiwan; 8 Kaohsiung Medical University, Kaohsiung, Taiwan; 9 School of Medicine, National Yang Ming Chiao Tung University, Taipei, Taiwan; 10 School of Medicine, Chung Shan Medical University, Taichung, Taiwan; 11 Division of General Surgery, Kaohsiung Chang Gung Memorial Hospital, Kaohsiung, Taiwan; 12 Department of Radiology, Changhua Christian Hospital, Changhua, Taiwan; 13 Division of Breast Surgery, Department of Surgery, Changi General Hospital, Singapore, Singapore; 14 Singhealth Duke-NUS Breast Centre, Singapore, Singapore

**Keywords:** Body mass index, Breast cancer, MRI, Axillary lymph node

## Abstract

**Background:**

We hypothesized that different BMI might have different impact on pre-operative MRI axillary lymph node (ALN) prediction accuracy and thereby subsequent surgical lymph node management. The aim of this study is to evaluate the effect of BMI on presentation, surgical treatment, and MRI performance characteristics of breast cancer with the main focus on ALN metastasis evaluation.

**Methods:**

The medical records of patients with primary invasive breast cancer who had pre-operative breast MRI and underwent surgical resection were retrospectively reviewed. They were categorized into 3 groups in this study: underweight (BMI < 18.5), normal (BMI of 18.5 to 24), and overweight (BMI > 24). Patients’ characteristics, surgical management, and MRI performance for axillary evaluation between the 3 groups were compared.

**Results:**

A total of 2084 invasive breast cancer patients with a mean age of 53.4 ± 11.2 years were included. Overweight women had a higher rate of breast conserving surgery (56.7% vs. 54.5% and 52.1%) and initial axillary lymph node dissection (15.9% vs. 12.2% and 8.5%) if compared to normal and underweight women. Although the post-operative ALN positive rates were similar between the 3 groups, overweight women were significantly found to have more axillary metastasis on MRI compared with normal and underweight women (50.2% vs 37.7% and 18.3%). There was lower accuracy in terms of MRI prediction in overweight women (65.1%) than in normal and underweight women (67.8% and 76.1%).

**Conclusion:**

Our findings suggest that BMI may influence the diagnostic performance on MRI on ALN involvement and the surgical management of the axilla in overweight to obese women with breast cancer.

## Background

Breast cancer is the most common cancer in women worldwide [1]. Body mass index (BMI) has become the most well-adopted index of body weight [2, 3] and obesity had been shown in many studies to be related to breast cancer incidence and outcome [4–6]. Furthermore, increase in the incidence of breast cancer-specific death was reported in obese ladies in a recent meta-analysis [7]. A previous large, population-based case–cohort study [8] also found that obesity appeared to influence breast cancer survival in part by greater tumor size, positive nodal status, and distant metastasis, with a 1.7-fold increased risk of stage III/IV disease in obese women compared to normal weight women.

Axillary lymph node (ALN) staging is an important part in the surgical management of breast cancer. Axillary nodal involvement is a well-known indicator of poor prognosis. Previously, axillary lymph node dissection (ALND) was the gold standard in determining the status of ALNs in patients with breast cancer. Sentinel lymph node biopsy (SLNB), which was associated with less morbidity, had gradually replaced ALND for surgical ALN evaluation in patients with early breast cancer [9–11]. ALND was performed if nodal metastasis was confirmed on SLNB or preoperative percutaneous biopsy. However, two landmark trials (NSABP-04 and ASCOG-Z0011) had caused a paradigm shift as these trials had shown that for breast cancer patients with T1–T2 stage and clinically negative lymph node (cN0), ALND could be safely omitted in selected patients having one or two sentinel lymph node (SLN) metastasis after breast preservation surgery and whole breast radiotherapy [12, 13]. Since ALND may not be necessary in women with metastatic axillary disease who meet the trial criteria, these studies had changed the role of pre-operative axillary imaging from identifying ALN metastasis to detecting patients with advanced (more than 2 metastatic LNs) or high-level axillary lymph nodes (metastasis in level II or III LNs). In other words, pre-operative axillary imaging plays an important role on identifying patients who are suitable for SLNB [14] or even omitting biopsy in some conditions [15, 16].

For pre-operative staging and evaluation of ALN status, both ultrasound and MRI are commonly used non-invasive modalities [17]. Previous study had shown the sensitivity of pre-operative ultrasound for detecting ALN metastasis was similar in obese and non-obese patients [18]. However, there was paucity of data regarding the effect of BMI on the performance of MRI in ALN evaluation. Hence, our analysis might provide a better understanding of the effect of BMI on axillary lymph node evaluation especially with pre-operative MRI.

Therefore, the aim of this study is to evaluate the effect of BMI on presentation, surgical treatment and MRI performance characteristics of breast cancer with the main focus on axillary lymph nodes metastasis evaluation. We hypothesized that different BMI might have different impact on pre-operative MRI axillary lymph node prediction accuracy and thereby subsequent surgical lymph node management.

## Methods

### Patients

**Fig. 1:**
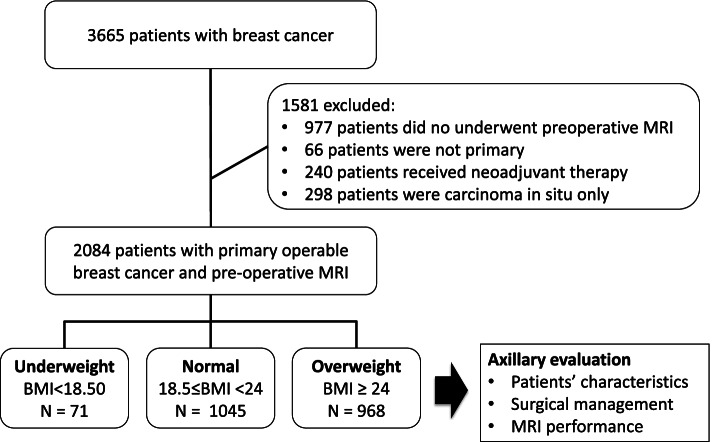
Flow chart of patient exclusion criteria

The study was approved by the Institutional Review Board of the Changhua Christian Hospital (CCH) (CCH IRB No. 141224 and No. 210519) and granted a waiver of informed consent. Women with invasive breast cancer and underwent surgical resection during the period of January 2010 to December 2020 were retrospectively recruited from the breast cancer database of CCH in Taiwan. Patients with non-primary breast cancer, carcinoma in situ only lesions, those who did not receive pre-operative MRI study, or patients who underwent neoadjuvant chemotherapy were excluded from the study. A flow chart of the patient selection process was shown in Fig. 1.

The clinicopathologic factors collected from the database include BMI, age, tumor location, biopsy method, types of breast operation, axillary LN staging method, pathologic tumor size, histology, tumor grade, status of estrogen receptor (ER), progesterone receptor (PR), human epithelial growth factor receptor 2 (HER-2) expression, and Ki-67 percentage, and axillary LN status.

BMI was calculated as weight in kilograms divided by height in meters squared (BMI = kg/m^2^). Using Taiwanese definition, BMI was categorized into four groups: underweight (BMI < 18.5), normal (BMI of 18.5 to 24), overweight (BMI of 24.1 to 26.9), and obese (BMI ≥ 27). Patients were categorized into 3 groups for the purpose of this study: underweight (BMI < 18.5), normal (BMI of 18.5 to 24), overweight (BMI > 24). We merged overweight and obese patients into one group because of the relatively small proportion of people with a BMI > 27 in our study cohort.

### Magnetic resonance imaging (MRI) protocol and evaluation

MR imaging was performed with a Siemens MAGNETOM Verio 3.0 Tesla MRI machine. All patients were imaged in the prone position with both breasts placed into a dedicated 16-channel breast coil. MR imaging protocols included the following: bilateral axial turbo-spin-echo fat-suppressed T2-weighted imaging (TR/TE 4630/70 ms; field of view 320 mm; slice thickness 3 mm; number of excitations 1), axial turbo-spin-echo T1-weighted imaging (TR/TE 736/9.1 ms; field of view 320 mm; slice thickness 3 mm; number of excitations 1), and diffusion weighted imaging (DWI) (TR/TE 5800/82 ms; field of view 360 mm; slice thickness 3 mm, with *b* values of 0, 400, and 800 s/mm^2^). Dynamic contrast enhanced MR images (DCE-MRI) were obtained with a three-dimensional fat-suppressed volumetric interpolated breath-hold examination (VIBE) sequence with parallel acquisition once before and five times after a bolus injection of gadobenate dimeglumine (0.1 mmol/kg). Both breasts were examined in the transverse plane at 60 s intervals in each phase of the dynamic studies. The dynamic MRI parameters were as follows: TR/TE 4.36/1.58 ms; field of view 320 mm; slice thickness 1 mm. The following criteria were used to identify suspicious metastatic lymph nodes: marked enhancement, loss of fatty hilum, cortical thickening (> 3 mm), round, or irregular shape [19, 20]. The whole-breast MRI readings were carried out by two experienced, board-certified breast radiologists (HKW, CYL, and WPW).

### Statistical analyses

Data were expressed as mean ± standard deviation for continuous variables. The Kolmogorov-Smirnov test demonstrated that the samples followed a normal distribution. The independent *t* test was used to compare continuous variables. Categorical variables were compared using the chi-square test or Fisher’s exact test, as appropriate.

The chi-square test was used to assess the associations between BMI and the evaluation of ALN metastasis on MRI. Final surgical histopathologic findings at either SLNB or ALND were used as reference standards for ALN evaluation. Diagnostic performance parameters (sensitivity, specificity, positive predictive value (PPV), negative predictive value (NPV), and accuracy) in each BMI subgroup were calculated for breast MRI regarding the surgical axillary LN status. True negative was defined as MRI showing negative ALN lymph nodes and surgical lymph node report also revealing no ALN metastasis. True positive was defined as MRI ALN evaluation as metastasis followed by final surgical outcome also confirming ALN metastasis. Sensitivity and specificity were defined as probabilities that in the case of positive ALN in the pathologic reports, MRI reported suspicious ALNs. Statistical analyses were performed by using Statistical Product and Service Solutions (SPSS) for Windows (Version 19.0, SPSS Inc., Chicago, IL).

## Results

### Patient and clinicopathological characteristics

According to the inclusion and exclusion criteria, a total of 2084 women with primary operable invasive breast cancer patients who received pre-operative breast MRI and surgical treatment were selected from CCH Breast Cancer Database. Among the 2084 women included in the study, 71 (3.4%) were considered as underweight (BMI < 18.5), 1045 (50.1%) as normal (18.5 ≤ BMI < 24), and 968 (46.5%) as overweight (BMI ≥ 24). The mean age of this cohort was 53.4 ± 11.2 years, and mean tumor size 2.3 ± 1.6 cm (Table 1). Overweight women were significantly older (56.1 ± 11.1 years) compared with normal (51.4 ± 10.7 years) and underweight women (47.6 ± 11.6 years, *p* < 0.01). The pathologic tumor size was significant larger in overweight women (*p* < 0.01). Underweight women (52.1%) tend to present with early stage (stage I) breast cancer at diagnosis if compared with overweight (34.5%) or normal (46.4%) women (*p* < 0.01). No significant differences between BMI groups were found for histological subtype, histologic grade, ER/PR/HER-2, and Ki-67 status (Table 1).

**Table 1:** Demographic and clinical characteristics by body mass index (BMI) subgroups

	Total *N* = 2084	Underweight BMI < 18.50 *N* = 71	Normal 18.5 ≤ BMI < 24 *N* = 1045	Overweight BMI ≥ 24 *N* = 968	*p* value
Age, years	53.4 ± 11.2	47.6 ± 11.6	51.4 ± 10.7	56.1 ± 11.1	< 0.01^†^
Location					0.36
Right	1006 (48.3)	38 (53.5)	515 (49.3)	453 (46.8)	
Left	1078 (51.7)	33 (46.5)	530 (50.7)	515 (53.2)	
Tumor size on MRI, cm	3.4 ± 1.9	3.0 ± 1.6	3.2 ± 1.8	3.6 ± 1.9	< 0.01^†^
Surgical method					0.52
Total mastectomy	928 (44.5)	34 (47.9)	475 (45.5)	419 (43.3)	
Partial mastectomy (BCS)	1156 (55.5)	37 (52.1)	570 (54.5)	549 (56.7)	
Specimen size, gm					
Total mastectomy	346.5 ± 200.1	153.7 ± 97.6	280.8 ± 140.8	466.9 ± 221.7	< 0.01^†^
Partial mastectomy (BCS)	62.7 ± 59.4	35 ± 22.8	53.1 ± 53.2	76.6 ± 65.6	< 0.01^†^
Surgical ALN staging method					< 0.01^†^
SLNB	405 (19.4)	11 (15.5)	182 (17.4)	212 (21.9)	
SLNB + ALND	1392 (66.8)	54 (76.1)	736 (70.4)	602 (62.2)	
ALND	287 (13.8)	6 (8.5)	127 (12.2)	154 (15.9)	
Pathologic tumor size, cm	2.3 ± 1.6	2.0 ± 1.5	2.2 ± 1.6	2.4 ± 1.6	< 0.01^†^
Pathologic stage					< 0.01^†^
I	856 (41.1)	37 (52.1)	485 (46.4)	334 (34.5)	
II	1000 (48.0)	25 (35.2)	464 (44.4)	511 (52.8)	
III	220 (10.6)	9 (12.7)	92 (8.8)	119 (12.3)	
IV	8 (0.4)	0	4 (0.4)	4 (0.4)	
Histological type	N/A = 63				0.73
IDC	1822 (90.2)	65 (91.5)	921 (90.6)	836 (89.5)	
ILC	97 (4.8)	3 (4.2)	50 (4.9)	44 (4.7)	
Others^a^	102 (5.0)	3 (4.2)	45 (4.4)	54 (5.8)	
Grade	N/A = 42				0.50
I	420 (20.6)	16 (23.2)	223 (21.7)	181 (19.1)	
II	1137 (55.7)	34 (49.3)	562 (54.7)	541 (57.2)	
III	485 (23.8)	19 (27.5)	242 (23.6)	224 (23.7)	
ER	N/A = 10				0.71
Positive	1714 (82.6)	61 (85.9)	856 (82.2)	797 (82.8)	
Negative	360 (17.4)	10 (14.1)	185 (17.8)	165 (17.2)	
PR	N/A = 9				0.77
Positive	1533 (73.9)	55 (77.5)	767 (73.6)	711 (73.9)	
Negative	542 (26.1)	16 (22.5)	275 (26.4)	251 (26.1)	
HER-2	N/A = 26				0.63
Positive	403 (19.6)	17 (23.9)	199 (19.3)	187 (19.6)	
Negative	1655 (80.4)	54 (76.1)	832 (80.7)	769 (80.4)	
Ki 67	N/A = 178				0.22
≦ 14	779 (40.9)	33 (48.5)	398 (41.9)	348 (39.2)	
> 14	1127 (59.1)	35 (51.5)	553 (58.1)	539 (60.8)	
Molecular subtype	N/A = 75				
Luminal A	767 (38.2)	26 (39.4)	401 (39.9)	340 (36.2)	0.61
Luminal B1	658 (32.8)	23 (34.8)	314 (31.2)	321 (34.2)	
Luminal B2	266 (13.2)	9 (13.6)	128 (12.7)	129 (13.8)	
HER-2(+)	151 (7.5)	6 (9.1)	78 (7.8)	67 (7.1)	
TNBC	167 (8.3)	2 (3.0)	84 (8.4)	81 (8.6)	

^†^Statistically significant difference

^a^*Others* = metaplastic carcinoma, *malignant phyllodes tumor,* papillary carcinoma

The mean total mastectomy specimen weight in the study cohort was 346.5 ± 200.1 g, and overweight women were significantly associated with larger specimen weight (466.9 ± 221.7) than normal (280.8 ± 140.8), and underweight (153.7 ± 97.6) patients (*p* < 0.01). Compared with underweight (52.1%) and normal (54.5%) women, overweight (56.7%) women had a higher chance of receiving breast conserving surgery (BCS). On the contrary, overweight women had higher rate of ALND as initial approach than normal and underweight women (15.9% vs. 12.2% and 8.5%, *p* < 0.01, Table 1).

### Diagnostic performance of prediction ALN status on MRI & impact of BMI

**Fig. 2:**
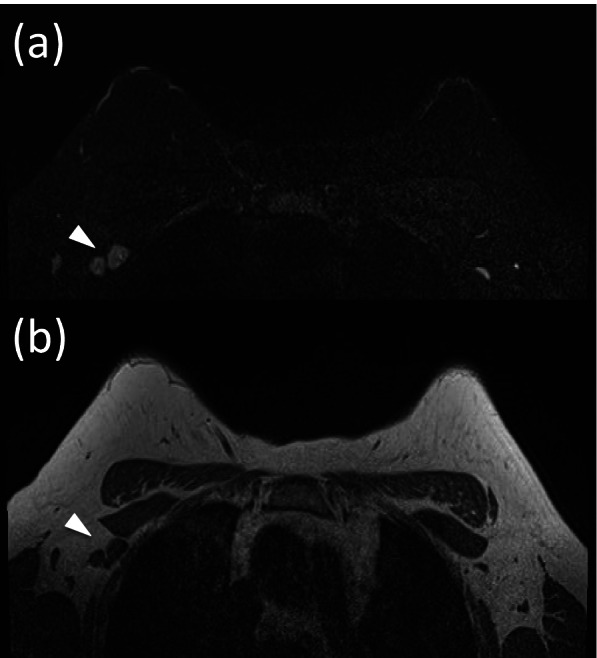
Breast MRI in a 40-year-old women in the overweight group (BMI 28.8) with invasive carcinoma in the right breast. **a** STIR axial image. **b** T1-weighted axial image shows suspicious lymph nodes in right axilla (arrowhead). But the pathology shows no metastatic lymph nodes are noted

**Fig. 3:**
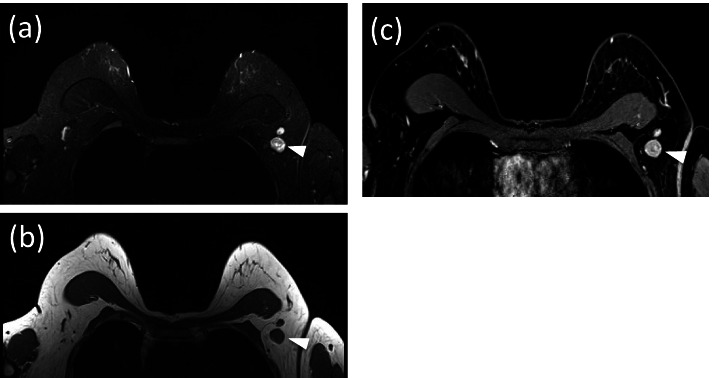
A female patient aged 38 years in the underweight group (BMI 17.7) with invasive ductal carcinoma in the left breast. MRI demonstrates enlarged lymph nodes (LN) in ipsilateral axilla (arrowhead). **a** STIR pulse sequence. **b** Pre-contrast T1-weighted pulse sequence. **c** The contrast-enhanced T1 fat-saturated pulse sequence. The pathology report confirmed axillary LN metastasis

**Fig. 4:**
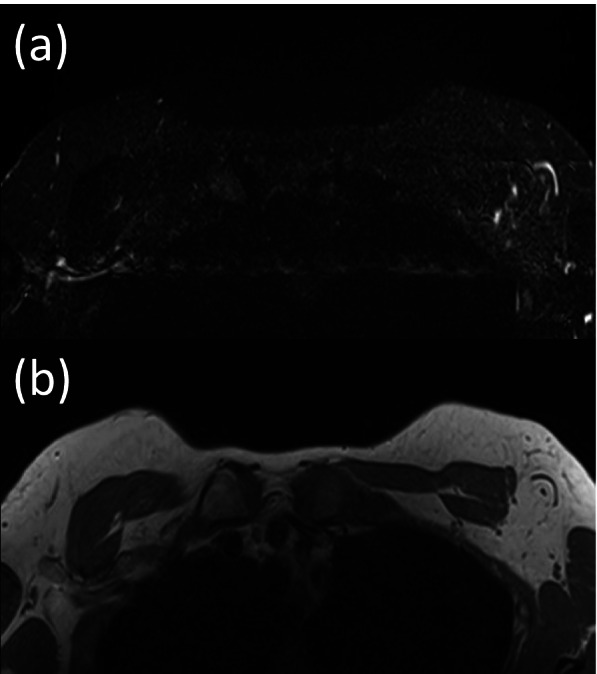
Breast MRI in a 40-year-old women in the normal group (BMI 21.2) with invasive carcinoma in the left breast. **a** STIR axial image. **b** T1-weighted axial image. No suspicious lymph nodes are seen. But the pathology shows several metastatic lymph nodes

A total of 2084 invasive breast cancer patients with pre-operative ALN MRI evaluation and post-operative ALN pathologic results were available for concordance analysis (Figs. 2, 3, and 4). Overweight women were significantly reported to have higher incidence of axillary metastasis on MRI if compared with underweight and normal weight women (50.2% vs 18.3% and 37.7%, *p* < 0.01, Table 2). The post-operative ALN positive rates were similar between all the 3 BMI subgroups (37.8%, 31%, 33.5%, *p* = 0.09).

**Table 2:** Diagnostic performance of MRI on axillary lymph node evaluation between BMI groups

	Total *N* = 2084	BMI < 18.5 *N* = 71	BMI 18.5 ~ < 24 *N* = 1045	BMI 24~ ***≥*** 35 *N* = 968	*p* value
ALN metastasis on MRI					< 0.01^†^
Yes	893 (42.9)	13 (18.3)	394 (37.7)	486 (50.2)	
No	1191 (57.1)	58 (81.7)	651 (62.3)	482 (49.8)	
ALN metastasis on pathology					0.09
Yes	738 (35.4)	22 (31.0)	350 (33.5)	366 (37.8)	
No	1346 (64.6)	49 (69.0)	695 (66.5)	602 (62.2)	
MRI diagnostic performance					
Sensitivity (%)	63.7	40.9	58.3	70.2	< 0.01^†^
Specificity (%)	68.6	91.8	72.7	62.0	< 0.01^†^
PPV (%)	52.6	69.2	51.8	52.9	0.46
NPV (%)	77.5	77.6	77.6	77.4	1.00
Accuracy (%)	66.8	76.1	67.8	65.1	0.10

^†^Statistically significant difference

*PPV* positive predictive value, *NPV* negative predictive value

For the concordance of ALN status between MRI and final pathology in the BMI subgroups analysis, overweight women significantly have the highest sensitivity (70.2%) but lowest specificity (62.0%). On the contrary, underweight women have the highest specificity (91.8%), and PPV (69.2%). NPV was similar between three groups: 77.6%, 77.6%, and 77.4% in underweight, normal, and overweight women respectively. Overall, the accuracy of breast MRI for detecting metastatic ALN was lower in overweight women (65.1%) than in normal and underweight women (67.8% and 76.1%, Table 2)

## Discussion

As the prevalence of obesity continues to increase in our population, understanding the effect of BMI on pre-operative clinical imaging of breast cancer is of paramount importance. Our current study showed that BMI influenced the diagnostic performance on MRI and the surgical management of ALN in overweight (BMI > 24) women with breast cancer and this was an important but rarely reported finding prior. We found that the accuracy of ALN prediction in breast MRI was lower in overweight women (65.1%) owing to the higher rate of false-positive prediction of pre-operative MRI imaging assessment.

ALN status is no doubt a critical component in surgical decision-making and in determining therapeutic strategies with significant impact on overall prognosis. Surgical axillary staging is still the gold standard for evaluating the status of ALN in breast cancer patients, be it either ALND or SLNB. Currently, SLNB had replaced ALND for surgical ALN evaluation in patients with clinically node negative breast cancer [21]. Meanwhile, pre-operative imaging assessment of the axilla has become increasingly more common in the current day breast cancer management. The value of ultrasound on axillary evaluation had been well documented [22–25]. However, the exact diagnostic performance of MRI for discriminating axillary lymph node involvement has inconsistent results reported by previous studies [26–35] (Table 3). As the results, to enhance the use of MRI on pre-operative axillary evaluation, identifying subgroup who would be benefit or less advantage from pre-operative MRI axillary evaluation is important. There were numerous studies evaluating the link between BMI and the risk, prognosis, and management of breast cancer [36, 37]. However, the impacts of BMI on pre-operative imaging assessment modalities were still not well established. Shah et al. showed that the sensitivity of pre-operative ultrasound assessment for detecting nodal metastasis was similar in newly diagnosed invasive breast cancer patients regardless of BMI [18]. On the contrary, we found that the sensitivity, specificity, and overall accuracy of ALN status as evaluated on MRI were significantly affected by BMI.

**Table 3:** Literature review of MRI diagnostic performance on axillary lymph node

Author	Journal/year	Patient numbers	Reference standard	Sensitivity (%)	Specificity (%)	NPV (%)	PPV (%)	Accuracy (%)
Yoshimura et al. [26]	Breast Cancer/1999	202	ALND	79.0	93.0	87.0	89.0	88.0
Kvistad et al. [27]	Eur Radiol/2000	65	ALND	83.0	90.0	90.0	83.0	88.0
He et al. [28]	Eur J Radiol/2012	136	ALND	33.3–86.5	95.2–98.2	1.9–16.7	66.7–82.6	18.5–96.2
Scaranelo et al. [29]	Radiology/2012	61	ALND/SLNB	88.4	82.4	94.7	69.4	85.0
Hwang et al. [30]	J Breast Cancer/2013	349	ALND/SLNB	47.8	88.7	82.6	60.2	77.9
Hieken et al. [31]	Surgery/2013	505	ALND/SLNB	54.2	78.2	75.7	57.7	69.7
Abe et al. [32]	Acad Radiol/2013	50	ALND/SLNB	60.0	79.0	81.0	59.0	74.0
An et al. [33]	Nuklearmedizin/2014	132	ALND	67.5	78.0	79.2	65.9	74
Hyun et al. [34]	Eur J Radiol/2016	425	ALND/SLNB	51.3	92.2	83.3	71.4	80.9
Barco et al. [35]	Clin Transl Oncol/2016	1351	ALND/SLNB	29.8	96.6	68.4	84.9	Not reported
Chen et al.	present study	2084	ALND/SLNB	63.7	68.6	77.5	52.6	66.8

*MRI* magnetic resonance imaging, *PPV* positive predictive value, *NPV* negative predictive value, *ALND* axillary lymph node dissection, *SLNB* sentinel lymph node biopsy

The sensitivity, specificity and accuracy of lymph node evaluation in overweight group (BMI > 24) were 70.2%, 62.0%, and 65.1%, respectively, while in the normal weight group they were 58.3%, 72.7%, and 67.8%, respectively. The lower specificity and accuracy were likely attributed to more false positive nodes on MRI in the overweight group. This result may imply that obesity could have a significant impact on nodal morphology features or size on MRI leading to limited diagnostic value. Alexander et al. found a highly significant association between increasing BMI and axillary LN dimensions, which was driven by expansion of the LN hilum secondary to fat infiltration [38]. Thus, using conventional MRI morphologic criteria to determine ALN metastasis may be limited. Other than morphologic features, functional and physiological assessments of the lymph nodes may be useful in detecting ALN metastasis. Buus et al. [39] found metastatic lymph node fat fraction on Dixon sequence were significantly lower than non-metastatic ipsilateral (*p* < 0.001) and contralateral lymph nodes (*p* < 0.001). Xing et al. [40] conducted a meta-analysis and found sensitivity of 0.86 and specificity of 0.86 for ADC values to discriminate between metastatic and non-metastatic axillary lymph nodes with AUC of 0.93. Superparamagnetic iron oxide (SPIO) MRI showed potential for a non-invasive modality of lymph node (LN) metastases evaluation [41]. However, whether BMI has impact on the diagnostic performance of these alternative methods remains unclear. Further studies combining additional sequence or specific contrast agent in the MRI assessment of the axilla in higher BMI breast cancer patients may be warranted.

Overweight to obese women, which was defined as BMI > 24 in current study, were usually correlated with larger breasts, and higher BCS rate compared with normal or underweight patients (Table 1). Similar to a previous study [42], our results showed that elevated BMI was not associated with a higher likelihood of ALN metastasis. However, we found a difference in surgical axillary staging between BMI categories, in that overweight to obese women had a higher proportion of receiving ALND as the initial approach. ALND is reserved as a secondary treatment procedure for patients with positive findings on SLNB or in clinically positive ALN patients. Higher ALND rate in the overweight group (BMI > 24) could be attributed to higher false-positive nodes on pre-operative breast imaging, like MRI.

Findings of suspicious ALN metastasis on imaging may subject patients to upfront ALND, especially if the patient did not receive neoadjuvant chemotherapy. Another possible explanation would be surgeons’ concern about the technical difficulty of ultrasound-guided fine needle aspiration/core needle biopsy and higher failure rate of SLNB in overweight/obesity patients. Although the precise reasons are largely unknown, several studies have reported lower SLN identification rates in obese women with dual-modality method for high BMI [43, 44]. Obese women who underwent ALND could potentially developed unfavorable effect after breast cancer surgery. Meijer et al. revealed ALND and high BMI are risk factors of developing breast cancer related lymphedema [45].

This study is limited by its retrospective nature and a single institution cohort which could lead to bias in treatment preferences. We aim to provide a general and complete information regarding the surgical management of breast tumor and BMI, and our patient cohort is large and representative of the contemporary distribution of BMI in Asian population. Our cohort of more than 2000 primary invasive operable breast cancer patients with pre-operative MRI evaluation and detailed post-operative pathologic reports enabled us to analyze the impact of BMI on the evaluation ALN status with MRI.

## Conclusions

Our study demonstrated that the diagnostic performance of MRI on pre-operative axillary lymph node assessment and hence surgical management was affected in overweight to obese women with breast cancer. Clinicians should therefore be cautious of using pre-operative MRI alone for the evaluation of ALN status, and specific strategies are needed to optimize the care of overweight to obese women with breast cancer.

## Data Availability

The datasets used and/or analyzed during the current study are available from the corresponding author on reasonable request.

## Abbreviations

BMI: Body mass index
ALN: Axillary lymph node
ALND: Axillary lymph node dissection
SLN: Sentinel lymph node
SLNB: Sentinel lymph node biopsy
ER: Estrogen receptor
PR: Progesterone receptor
HER-2: Human epithelial growth factor receptor 2
PPV: Positive predict value
NPV: Negative predictive value
